# Knockout of CXCR5 increases the population of immature neural cells and decreases proliferation in the hippocampal dentate gyrus

**DOI:** 10.1186/1742-2094-11-31

**Published:** 2014-02-17

**Authors:** Michael J Stuart, Frances Corrigan, Bernhard T Baune

**Affiliations:** 1 Discipline of Psychiatry, University of Adelaide, Adelaide SA 5005, Australia; 2 School of Medicine, James Cook University, Townsville QLD 4811, Australia; 3 Discipline of Psychiatry, School of Medicine, University of Adelaide, Adelaide SA 5005, Australia

**Keywords:** CXCR5, CXCL13, Chemokine, Proliferation, Stem cell, Progenitor cell, Hippocampus, Depression, Cognition

## Abstract

**Background:**

The process of neurogenesis in which new neurons are generated by proliferation and differentiation of neural stem/progenitor cells (NSCs/NPCs) has been a topic of intensive recent investigation. Investigations of the factors which regulate this process have recently begun to include immune factors including immune cells and cytokines, however the class of immune proteins designated as chemokines have been relatively neglected. Increasing evidence for novel brain-specific mechanisms of chemokines beyond their classical chemotactic functions has suggested that they may play a role in the regulation of NSC/NPC biology.

**Methods:**

We have investigated the role of the chemokine receptor CXCR5 (ligand is CXCL13) in the activity of these cells through neurobiological and behavioural analysis of CXCR5-deficient mice (CXCR5^-/-^). These investigations included: immunohistochemistry for the markers Ki67, nestin, doublecortin, and IBA-1, neurosphere assays, and the baseline behavioural tests: open field test and sucrose preference test.

**Results:**

We observed a significant increase in doublecortin and nestin staining in the hippocampal dentate gyrus (*P* = 0.02 and *P* = 0.0008, respectively) of CXCR5^-/-^ animals as compared to wild-type controls. This was accompanied by a decrease in Ki67 staining subgranular zone (*P* = 0.009). Behavioural correlates included a significant increase in baseline locomotor activity in an open field test (*P* <0.00018) and a decrease in stress reactivity in that test (*P* = 0.015). Deficiency in CXCR5 was not associated with alterations in hippocampal microglial density, microglial activation or systemic cytokine levels, nor with loss of NSC/NPC populations in the neurosphere assay.

**Conclusions:**

These findings are the first to describe a brain-specific function of CXCR5 under physiological conditions. CXCR5 reduces maintenance of immature neural cell populations and enhances proliferation of subgranular zone cells in the hippocampal dentate gyrus, however the mechanism of these effects remains unclear. Further research into the regulation of NSC/NPC activity should consider investigation of CXCR5 and other chemokines which may be relevant to the pathophysiology of psychiatric disorders including depression, anxiety and cognitive impairment/dementia.

## Background

The process of neurogenesis in which new neurons are generated by proliferation and differentiation of neural stem/progenitor cells (NSCs/NPCs) has been a topic of intensive investigation in recent years. This process has been demonstrated to persist into adulthood in many species - including humans where it is believed to be localised to several discrete anatomical regions including the subventricular zone (SVZ) and subgranular zone (SGZ) of the hippocampal dentate gyrus [1]. The latter region is of particular interest to the study of human health and disease given its key functions in learning and memory [2]. Indeed, the dysregulation of hippocampal neurogenesis may be relevant to the pathogenesis of several disease processes of immense public health significance including depressive disorders and the spectrum of mild cognitive impairment to Alzheimer’s type dementia [3,4]. This process may also be relevant to the mechanism of action of commonly prescribed antidepressant medications including selective serotonin and/or noradrenaline reuptake inhibitors [5]. An enhanced understanding of the mechanisms by which NSC/NPC proliferation and differentiation are regulated may therefore provide a basic science platform for further translational work to provide novel diagnostic and therapeutic tools for these disorders which are lacking in the field.

The regulation of NSC/NPC activity has been shown to include a complex interplay of many biological systems including neurotrophic factors (for example, Brain Derived Neurotrophic Factor (BDNF)) [6], neurotransmitters (for example, Noradrenaline (NA)) [7], immune cells (for example, Th_1_/Th_2_/T_reg_ balance) [8,9] and soluble immune factors (for example, Interferon-γ) [10]. It is notable that the investigation of soluble immune factors to date has primarily focused on pro-inflammatory cytokines and other classes, including chemokines, have been relatively neglected. Emerging evidence has begun to describe novel actions of the immune proteins designated as chemokines in the central nervous system (CNS) beyond their classical chemotactic functions. These may include regulating the infiltration and activation states of central and peripheral immune cells, regulation of neuroendocrine functions, pre- and post-synaptic modulation of neurotransmitter systems, and possibly direct neurotransmitter-like effects [11,12]. Recently the chemokine CX_3_CL1 has been demonstrated to be a critical mediator of the beneficial effects of exercise on hippocampal neurogenesis via regulation of microglial phenotype, therefore it may be possible that other chemokines might have direct or indirect effects on the regulation of neurogenesis including via microglia [13].

We have chosen to investigate the effects of the chemokine receptor CXCR5 (receptor for CXCL13) on hippocampal neurogenesis. In the periphery this protein is known to play a role in directing the migration of lymphocytes, particularly B cells [14,15]. Modulation of the systemic immune response may be relevant to the regulation of hippocampal neurogenesis, for example in the context of psychiatric disorders [16,17]. It has also been shown to be widely expressed in the CNS including on microglia, astrocytes, mature neurons and NPCs; however, its mammalian neurobiological significance under physiological conditions remains unknown [18,19]. Recent work in zebrafish has demonstrated that CXCR5 is expressed on radial glia cells (neural progenitor cells), and is permissive of proliferation of these cells [20]. Therefore CXCR5 has the potential to influence hippocampal neurogenesis by CNS-specific or systemic immune mechanisms. We aim to investigate the roles of CXCR5 on the activity of hippocampal NSC/NPCs, whether any effects on those cells are mediated by alterations in systemic or CNS-resident immune factors, and the behavioural correlates of these effects. This will be the first study to investigate such effects.

## Materials and methods

The experimental design utilises a simple subtractive methodology comparing knockout mice for CXCR5 (designated CXCR5^-/-^) to wild type (WT) in all experimental steps.

### Animals and ethics

The CXCR5^-/-^ animals and WT controls for the study were acquired from the Jackson Laboratories ( http://jaxmice.jax.org/strain/006659.html). These animals are on a C57/BL6J background. They were housed in approved conditions on a 12-hour light/dark cycle in individually ventilated cages with three to five animals to each cage. Food and water were provided *ad libitum*. Handling occurred twice per week when each animal was weighed and assessed for general health. All care occurred under the oversight of a veterinarian.

Cohorts of 11 male and female (3 male, 8 female, total n = 11) animals of each genotype were aged to 10 weeks before commencing behavioural studies and were sacrificed by 11 weeks of age for immunohistochemistry (5 female, 1 male, total n = 6) and protein analysis (3 female, 2 male, total n = 5). Cohorts of 10 male animals of each genotype were sacrificed at 6 weeks of age for tissue use in the neurosphere assay, as at this age adult-derived hippocampal neurospheres are maximal [21]. All animals were sacrificed by cervical dislocation by a trained operator.

This study received ethics approval from the University of Adelaide animal ethics committee.

### Neurosphere assay

Primary neurospheres from SVZ and hippocampus were separately generated according to previously published protocols [13,22]. In brief: hippocampal or SVZ tissue was digested by incubation in a mixture containing 0.1% papain (Worthington Biochemical Corporation) and 0.1% DNaseI (Roche Australia) in HBSS (Thermo Scientific) for 16 min at 37°C, titurating twice during the incubation period. Next, the tissue was centrifuged at 750 rpm for 5 min, after which the pellet was resuspended and washed twice in 2 mL of neurosphere growth medium: mouse NeuroCult NSC basal medium containing mouse NeuroCult NSC proliferation supplements (Stem Cell Technologies), 2 μg/mL heparin, 20 ng/mL purified mouse epidermal-like growth factor (BD Biosciences) and 10 ng/mL recombinant bovine fibroblast growth factor02 (Roche). Cells were plated at a density of two hippocampus or SVZ per 96-well plate (BD Biosciences) with 200 μL of neurosphere growth medium per well. For depolarisation KCl was added to half the primary cultures of each hippocampus for a final concentration of 15 mM. SVZ cultures were incubated for 7 days and hippocampal cultures for 10 days in humidified 5% CO_2_. Neurospheres were then counted using a standard light microscope with an eyepiece graticule. Small neurospheres (likely derived from NPCs) were defined as ≥50 μm diameter and large neurospheres (likely derived from putative NSCs) were defined as ≥250 μm diameter [21]. Cultures were not passaged further. The operator was blinded to the genotype for counting.

### Immunohistochemistry

At time of sacrifice brain tissue was collected from six animals (5 females, 1 male) of each genotype (n = 6). These mice were perfused with a 10 mL of 10% formalin solution through the left ventricle followed by retrieval of brain tissue and storage in a 10% formalin solution. Brains were then embedded in paraffin prior to sectioning, with a series of nine 5 μm sections collected at 200 μm intervals throughout the thickness of the hippocampus. Immunostaining was then conducted using antibodies for Ki67 (Abcam), doublecortin (Millipore), Nestin (Abcam)s and IBA-1 (Santa-Cruz). Following de-waxing and dehydration, endogenous peroxidase activity was blocked by incubation with 0.5% hydrogen peroxide in methanol for 30 min. Slides were then washed in 2 × 3 min in phosphate buffered saline (PBS) before antigen retrieval retrieved by heating at close to boiling point for 10 min (TRIS for doublecortin, citrate for others). Once the slides had cooled below 40°C they were washed with PBS before being blocked with 3% normal horse serum in PBS for 30 min. The appropriate primary antibody was applied to the slides which were left to incubate overnight (Ki67 1:2,000, doublecortin 1:8,000, Nestin 1:500, IBA-1 1:1,000). The next day slides were washed in 2 × 3 min of PBS before the appropriate species of IgG biotintylated antibody was added for 30 min (Dako). After a further PBS wash, slides were incubated with streptavidin peroxidase conjugate for 60 min followed by another rinse with PBS. The immunocomplex was then visualised with precipitation of DAB (Sigma D-5637) in the presence of hydrogen peroxide. Slides were washed to remove excess DAB and lightly counterstained with haematoxylin, dehydrated and mounted with DePeX from histolene.

Slides were subsequently digitally scanned using the Hamamatsu NanoZoomer and examined using the associated NDP.view2 software (Hamamatsu). Sequential images were captured of the hippocampus and exported for cell counting within Image J 1.46 (NIH) where the grid and manual cell counter features were utilised. In all instances the hippocampus or dentate gyrus were counted in their entirety, bilaterally. Area of each region was measured in NDP.view2 and results were expressed as cellularity in cells per mm^2^. The operator was blinded to the genotype of the slides during the slide analysis process.

### Cytometric bead assay

At time of sacrifice blood was collected from five animals of each genotype (3 females, 2 males) by means of cardiac puncture and serum was extracted by spinning blood down at 2,500 rpm for 15 min. Serum was stored at -80°C until analysis. Serum cytokine levels were measured using the BD Cytometric Bead Array (CBA) Mouse Inflammation Kit for the cytokines IL-6, IL-10, MCP-1, IFN-γ, TNF-α and IL-12p70 according to the manufacturer’s instructions.

### Open field test

For the open field and hole-board tests, mice were placed into the same brightly lit square arena, 40 × 40 cm, with clear walls 35 cm high. In the open field test, mice were placed in to the arena for 5 min according to published protocols [23,24]. The floor was divided into inner and outer zones. Time spent in each zone was measured as an indication of anxiety-like behaviour, and total distance travelled was measured as an indication of baseline locomotor activity.

### Saccharin preference test

Mice were trained for the saccharin preference test with a continuous two-bottle choice of water and 0.1% saccharin for 48 h. For this period, mice were moved to individual cages which could hold two bottles and the bottles were switched at 24 h to control for left-right preference. Immediately after this training period, the test was conducted. The test involved mice being moved to individual cages and given the two-bottle choice of water and 0.1% saccharin for 24 h. This protocol has been adapted from several previously published protocols [25,26]. Preference was calculated as: saccharin preference *%* = (saccharin cons./(saccharin cons. + water cons.)) × 100.

### Statistical analysis

All data were analysed using SPSS version 21 software (IBM). All data passed normality testing using the Shapiro-Wilk test. Comparisons between genotypes for all data were made using independent samples T tests. For all results, a *P* value of ≤0.05 was considered significant. All data presented in graphs are mean values ± standard error of the mean (SEM).

## Results

### CXCR5 deficiency is permissive of DCX + and Nestin + population maintenance and suppresses subgranular zone cell proliferation

**Figure 1:**
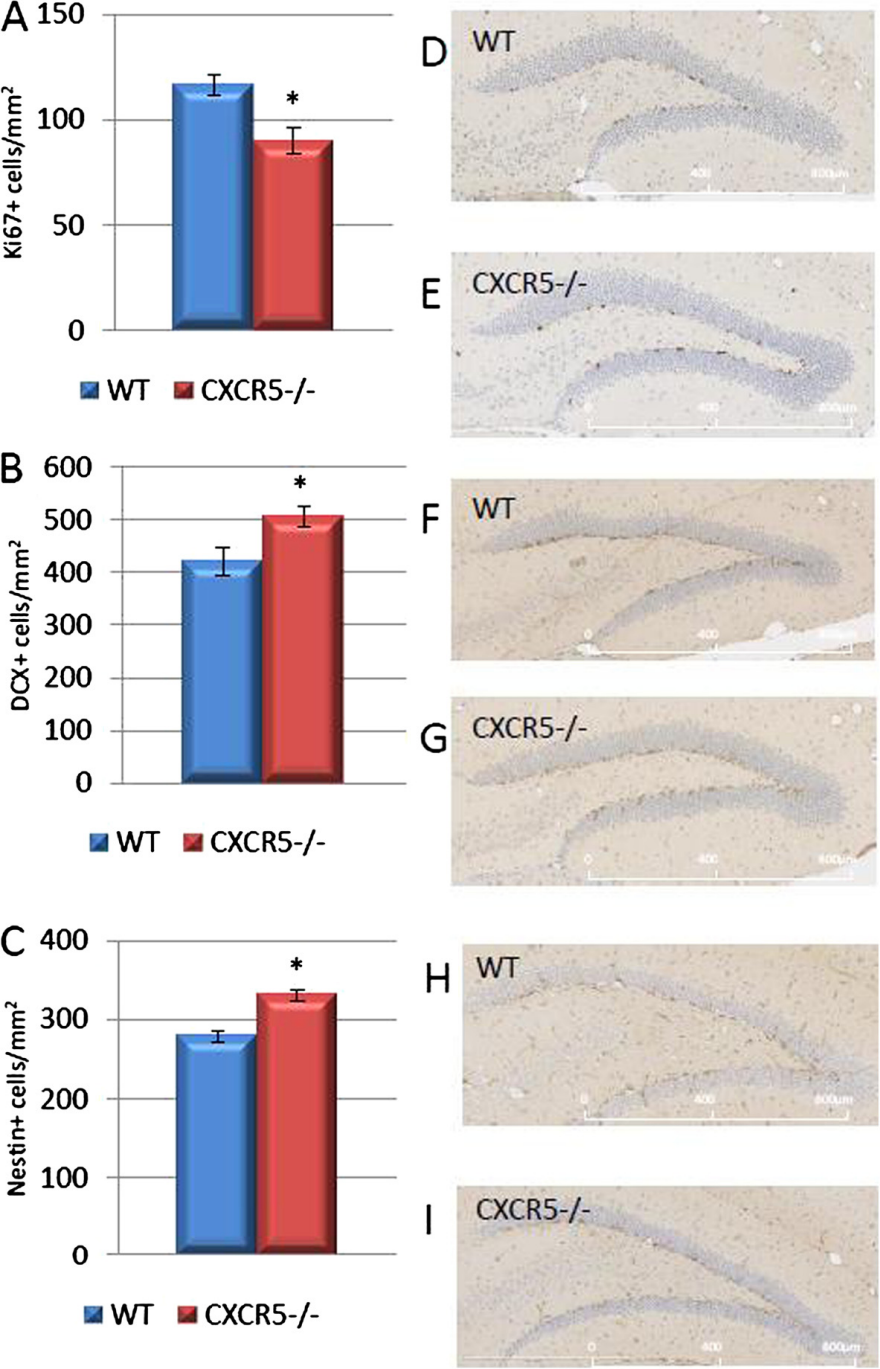
**Effect of CXCR5 deficieny on neurogenesis in the adult dentate gyrus. (A)** In CXCR5^-/-^ animals the number of Ki67 positive cells was significantly reduced compared to WT controls (*P* = 0.009; n = 6 per group). **(B)** In CXCR5^-/-^ animals the number of DCX positive cells was significantly increased compared to WT controls (*P* = 0.02; n = 6 per group). **(C)** In CXCR5^-/-^ animals the number of Nestin positive cells was significantly increased compared to WT controls (*P* = 0.0008; n = 6 per group). Representative images of Ki67 **(C, D),** DCX **(E, F)** and Nestin **(H, I)** staining.

We investigated whether CXCR5 deficiency might impact upon hippocampal neurogenesis in 11-week-old (adult) mice. In order to assess this, we stained hippocampal sections for the proliferation marker Ki67, Nestin doublecortin (DCX), markers of immature neuronal cells. We counted only positive cells within the subgranular zone for Ki67. Within this subregion Ki67 positive cells are likely to represent proliferative activity of NPCs, and Nestin/DCX the immature cells of neuronal lineage which are generated by the differentiation and migration of those cells [27]. We found that CXCR5 deficiency was permissive of increased density of DCX positive cells in the granular and subgranular zones of the dentage gyrus (505 ± 19.2 *vs.* 420 ± 25.9; n = 6 per group; *P* = 0.02). A similar trend was observed in nestin positive cells (331 ± 8.2 *vs.* 280 ± 7; n = 6 per group; *P* = 0.0008) (Figure 1). This implies that CXCR5 signaling may impair the maintenance of these populations. Additionally, we demonstrated that CXCR5 deficiency suppressed the number of Ki67 positive cells in the subgranular zone as compared to WT controls (90.0 ± 6.3 *vs.* 116.3 ± 5.1; n = 6 per group; *P* = 0.009). This may represent indirect suppression of a compensatory mechanism which would act to maintain DCX and Nestin positive cells under physiological conditions, or a direct effect by which CXCR5 (known to be expressed on NSC/NPC [19]) would support proliferation of these cells. Taken together these data are most suggestive that CXCR5 may exert a detrimental effect on maintenance of Nestin and DCX positive cell populations - for example through a pro-apoptotic effect - requiring enhanced proliferation of subgranular zone cells to compensate. A further possibility is that CXCR5 may support differentiation further down a neuronal lineage, thereby 'draining’ the pools of DCX and Nestin positive cells and requiring an enhancement of subgranular zone cell proliferation (Ki67 positive) to replace the pool. An alternative explanation, in which CXCR5 impairs differentiation of proliferating cells into a neuronal lineage is less likely as this effect would not be expected to simultaneously enhance the proliferation of the subgranular zone precursor/stem cells.

### No effect of CXCR5 deficiency on neural stem/progenitor cell populations

**Figure 2:**
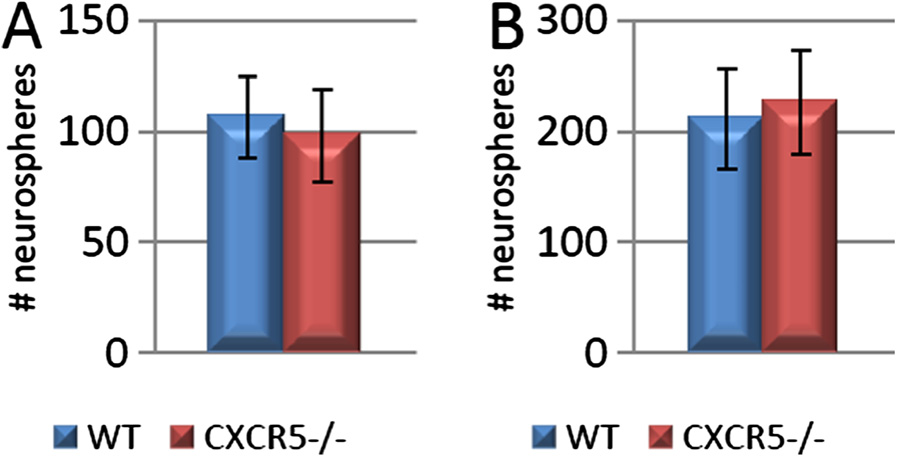
**CXCR5 deficiency does not alter hippocampus-derived neural precursor activation. (A)** No significant difference in ≥50 μm neurosphere formation between CXCR5^-/-^ animals and WT controls under basal culture conditions (*P* >0.05; n = 10 per group). **(B)** No significant difference in ≥50 μm neurosphere formation between CXCR5^-/-^ animals and WT controls under KCl depolarised conditions (*P* >0.05; n = 10 per group).

To further interpret the observed *in-vivo* effects of the CXCR5 deficiency we also analysed the *in-vitro* activity of several subgroups of NPC/NSCs via the neurosphere culture method. We considered separately the populations of small (≥50 μm) and large (≥250 μm) hippocampus derived neurospheres both under basal culture conditions and the latent populations which are responsive to depolarisation with KCl. Previous studies have described the properties of these populations to include cells of both NPC and in the case of KCl responsive large spheres NSC phenotype [21]. In this study we detected no significant effects of CXCR5 deficiency on any of these hippocampal populations in number or size (n = 10 per group; all *P* >0.05) (Figure 2). We also detected no significant difference in SVZ derived neurospheres (75.5 ± 12.5 *vs.* 110.9 ± 15.5; n = 10 per group; *P* = 0.1) (data not shown). Considered in parallel with the above data, this suggests that the observed reduction in Ki67 staining seen in CXCR5^-/-^ animals is not due to an absolute loss of NPC/NSC populations as these are still reactive to appropriate *in-vitro* stimulus. This implies that CXCR5 signalling is not required for maintenance of the proliferative capability of latent NPC/NSC populations.

### No effect of CXCR5 deficiency on microglial density, microglial activation or systemic cytokine levels

**Figure 3:**
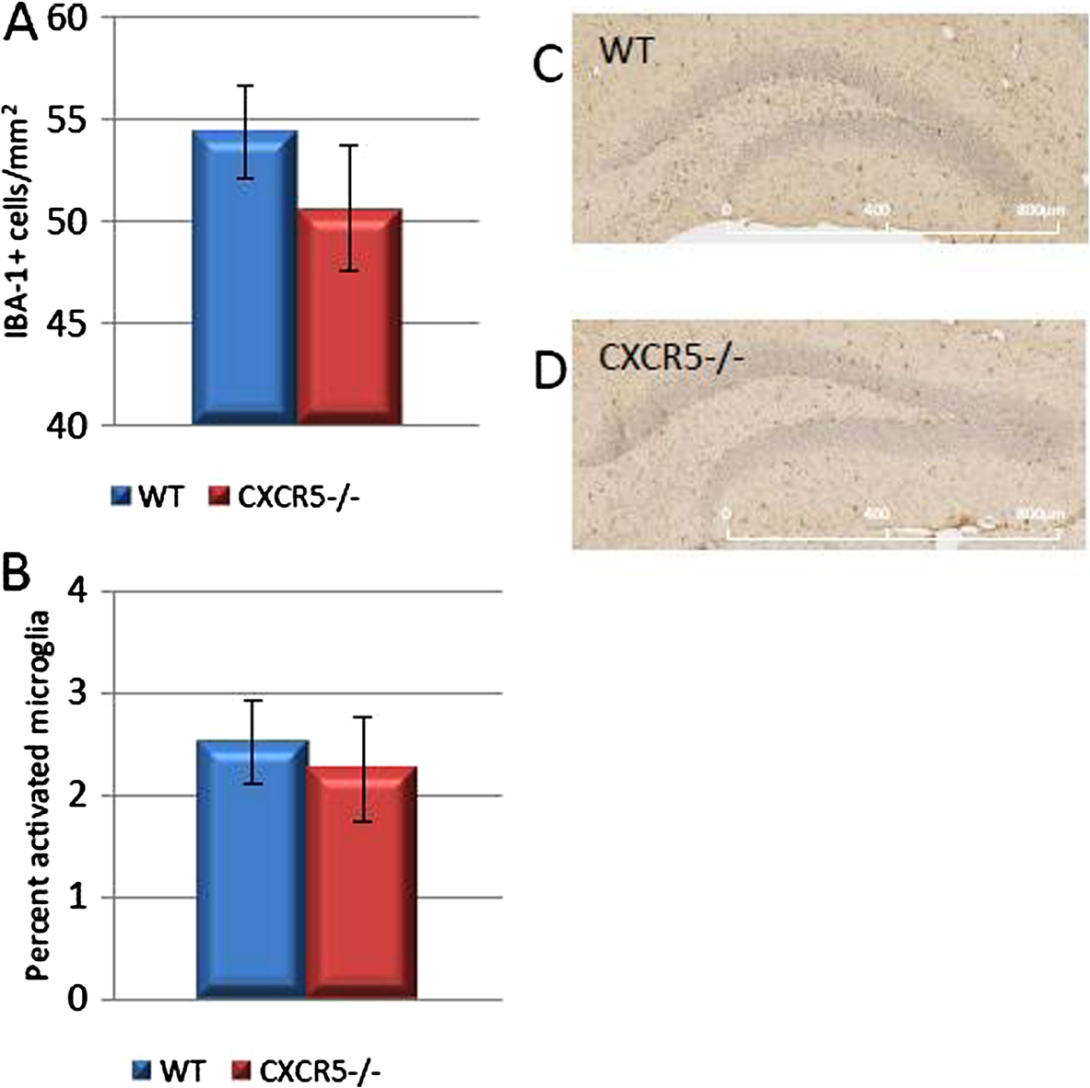
**No effect of CXCR5**^**-/- **^**on hippocampal IBA-1 staining. (A)** No difference in hippocampal microglia between CXCR5^-/-^ and WT control animals (*P* >0.05; n = 6 per group). **(B)** No difference in percentage of activated hippocampal microglia between CXCR5^-/-^ and WT animals (*P* >0.05; n = 6 per group). **(C, D)** IBA-1 stained WT/CXCR5^-/-^ dentate gyrus representative images, respectively.

To investigate the mechanisms by which CXCR5 deficiency may produce a deficit in the number of Ki67 positive cells we investigated several indices of the CNS specific and systemic immune response which are known to influence normal neurogenesis. We investigated the density of IBA-1 positive cells (microglia) in the hippocampus, as depletion of these cells is known to impair neurogenesis [13]. We detected no significant effect of CXCR5 deficiency on density of these cells in this study (50.7 ± 3.1 *vs.* 54.4 ± 2.3 cells/mm^2^; n = 6 per group; *P* = 0.37) (Figure 3). Furthermore, we analysed the percentage of activated (reactive) microglia on the basis of their classically described morphology: large soma, short branches and a 'bushy’ appearance [28]. We detected no difference in the percentage of activated microglia (2.25 ± 0.4 *vs.* 2.52 ± 0.52%; n = 6 per group; *P* = 0.69) (Figure 3).

We also assayed the levels of systemic cytokines through use of a cytometric bead assay on serum as several cytokines are known to enhance or impair neurogenesis (for example, Interferon-γ) [10]. There were no significant differences between genotypes in serum levels of the cytokines IFN-γ, IL-6, IL-10, MCP-1, TNF-α or IL-12p70 (all *P* >0.05) (data not shown). These data suggest that the observed effects of CXCR5 on Ki67, Nestin and DCX cell populations are not mediated by alterations inmicroglial population or gross disruption of soluble immune factors which may ingress from the systemic circulation. Under these non-immunologically challenged experimental conditions, and in the absence of gross disruption of systemic or glial immune function we would not anticipate alteration in CNS-specific cytokine profile.

### CXCR5 deficiency results in an increase in baseline locomotor activity

**Figure 4:**
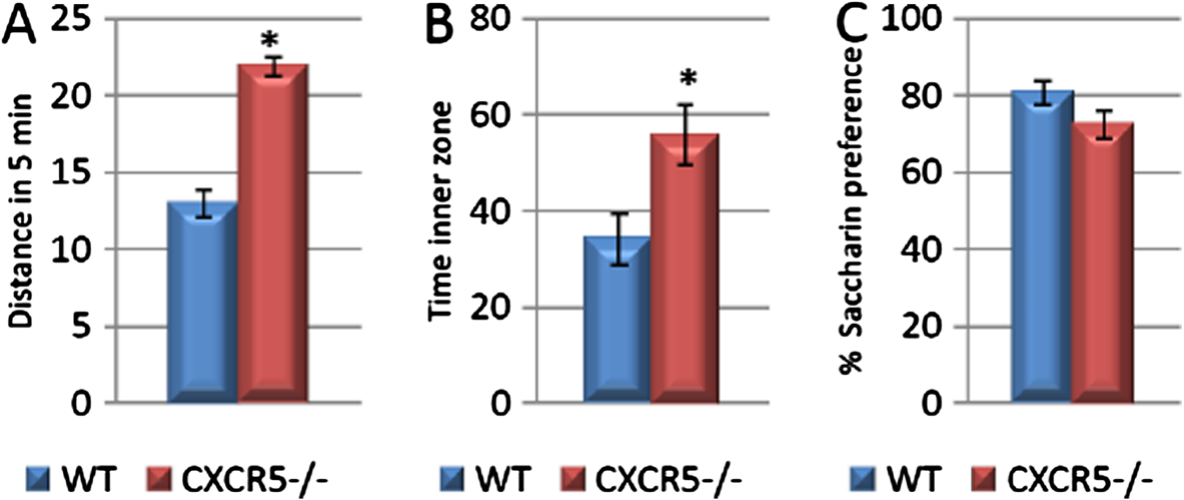
**CXCR5**^**-/- **^**is associated with increased baseline locomotor activity and decreased anxiety-like behaviour but not anhedonia-like behaviour. (A)** CXCR5^-/-^ is associated with increased locomotor activity over 5 min in the open field test as compared to WT controls (* *P* <0.0001; n = 11 per group). **(B)** CXCR5^-/-^ is associated with increased time spent in the inner zone of the open field test as compared to WT controls (* *P* = 0.015; n = 11 per group). **(C)** No difference in saccharin preference between CXCR5^-/-^ and WT animals (*P* = 0.098; n = 11 per group).

We next aimed to investigate the behavioural consequences of CXCR5 deficiency. A useful initial test is the open field test which provides information on baseline locomotor activity which may be a significant confounder for further behavioural testing [29]. In our study we found that CXCR5 deficiency was associated with a two-fold increase in baseline locomotor activity as assessed by distance travelled during the test (21.9 ± 0.6 *vs.* 13.0 ± 0.9; n = 11 per group; *P* <0.0001) (Figure 4). We also found an increase in time spent in the inner two thirds of the open field by CXCR5 deficient animals which may be interpreted as a reduction in stress responsiveness (55.9 ± 6.2 *vs.* 34.1 ± 5.4; n = 11 per group; *P* = 0.015), however such an interpretation should be regarded with caution given the overall increase in locomotor activity (Figure 4A, B). In view of the result on locomotor activity which would make it difficult to interpret the results of conventional hippocampus-dependent learning and memory tasks (for example, Barnes Maze or Morris Water Maze [30]), we have pursued behavioural testing which did not require locomotor activity.

### CXCR5 deficiency does not alter anhedonia-like behaviour

Although the mechanistic relevance of hippocampal neurogenesis to depression or depression-like behaviours remains unclear, it is well established that in many animal models of stress and/or depression-like behaviour these behaviours are correlated with impairments in neurogenesis, and certain antidepressant medications are associated with increases in neurogenesis [5]. We therefore investigated the effects of CXCR5 deficiency on the saccharin preference test which is a well established model of anhedonia-like behaviour and does not require locomotor activity [31]. All animals consistently demonstrated a preference for the saccharin-sweetened solution during training (data not shown), however during testing there was no significant effect of CXCR5 deficiency on saccharin preference as compared to WT controls (72.6 ± 3.5 *vs.* 80.65 ± 3.0; n = 11 per group; *P* = 0.1) (Figure 4C). Interestingly, these data suggest a non-significant trend toward an anhedonia-like phenotype of CXCR5 deficient mice - the opposite of what would be expected if depression-like behaviours were associated with impaired neurogenesis.

## Discussion

This is the first study to suggest that CXCR5 may impair the maintenance of hippocampal neuroblast and neuronal precursor cell populations, while increasing the proliferation of (Ki67 positive) cells in the subgranular zone of the dentage gyrus, however the mechanism for this and the consequences for learning and memory function remain uncertain. This result, obtained in mice is somewhat in contrast to recent results suggestive of a role of CXCR5 in supporting proliferation of immature neuronal cells in zebrafish [20]. There are several possible explanations for these results. Perhaps the most likely is that CXCR5 may exert a detrimental effect on the hippocampal neuroblast precursor cell populations - perhaps through an inflammatory or pro-apoptotic mechanism. While our preliminary investigations here have not found any gross alteration in CNS specific or systemic immune function, we cannot exclude such an effect. The alternative possibility that CXCR5 may affect the differentiation of these cells also cannot be excluded with our data. Future studies such as *in-vitro* addition of recombinant CXCL13 to neurosphere cultures may shed further light on this possibility.

The behavioural significance of these neurobiological differences also remains to be elucidated. Although we noted an increase in immature cells of a neuronal lineage, CXCR5 deficiency did not significantly influence anhedonia-like behaviour. In the presence of a difference in DCX staining, it may be expected that these mice would demonstrate differences in hippocampus dependent learning and memory tasks - however we were unable to assess this on commonly used behavioural tests given the confounding effects of the increased baseline locomotor activity of the transgenic animals. In this situation it may be relevant to apply novel behavioural testing strategies which are not reliant on locomotor activity such as mouse touchscreen tasks analogous to components of widely used human batteries (for example, Cambridge Neuropsychological Test Automated Battery (CANTAB)) [32].

This early result should trigger further investigation of the neurobiological roles of this chemokine. Several lines of investigation may provide further useful insight. First, our study has considered relatively young (<3-month-old) mice under basal conditions where no stimulus to increase the demand for neurogenesis has been applied. If CXCR5 is involved in apoptotic processes or the differentiation of cells to a neuronal lineage application of pro-apoptotic or pro-neurogenic stimulus may assist to differentiate these effects. Many options of pro-neurogenic stimulus are available in a spectrum from exercise to the robust stimulation associated with seizure models [13,21,33]. Conversely, pro-apoptotic or anti-neurogenic stimulus may include the analysis of aged mice (for example, 9 months of age) or the application of environmental stressors such as a chronic unpredictable mild stress paradigm [4,23]. Finally, further investigation of the mechanisms by which CXCR5 modulates the proliferation of subgranular zone cells may assist to elucidate the role of this chemokine in the complex interrelated mechanisms which collectively regulate the activity of NSC/NPCs. These investigations should seek to clarify whether the influence of CXCR5 is via direct effects of this receptor as expressed on NPCs or whether it is via indirect effects of the receptor expressed on other CNS resident cells including microglia [18,19]. This may be clarified by the addition of the recombinant ligand for this receptor (CXCL13) to neurosphere cultures which have been selectively depleted for other cell types following established protocols [13]. Furthermore, the measurement of other soluble factors in brain tissue including cytokines and neurotrophic factors may provide more comprehensive insight into the effects of this knockout on the local hippocampal milieu.

In a broader context the investigation of novel mechanisms which may regulate the activity of NSC/NPCs may provide insight into not only the physiology of normal hippocampal function, but also the pathophysiology of depressive disorders, anxiety disorders and disorders of cognitive function [5,34]. Chemokines have a particularly strong rationale for further investigation as mediators of NSC/NPC activity as many members of this class have been found to be differentially expressed in clinical studies of patients with the aforementioned disorders [35,36]. With further translational development chemokines and receptors may prove to be clinically relevant targets for novel diagnostic or therapeutic strategies in the management of these disorders.

## Conclusions

This is the first study to demonstrate a role for CXCR5 in mammalian hippocampal neurobiology. CXCR5 reduces maintenance of immature neural cell populations and enhances proliferation of subgranular zone cells in the hippocampal dentate gyrus, however the mechanism of these effects remains unclear. Further research will be required to independently replicate these findings and further investigate both the mechanisms of this effect and its cognitive-behavioural correlates. Such investigations should include a TUNEL assay, BrdU uptake staining and *in-vitro* differentiation assays. Further elucidation of the role of CXCR5 as a regulator of hippocampal neurogenesis may provide novel insights into the CNS specific functions of chemokines and stimulate further investigation of this relatively neglected family of immune proteins.

## Competing interests

The authors declare that they have no competing interests.

## Authors’ contributions

MS and BB conceived and designed the study. MS conducted the laboratory work and wrote the initial draft. FC contributed to revisions and additional experimentation as requested by peer reviewers. BB and MS revised the draft. All authors have read and approved the final submission.
